# Silencing of Chitin-Binding Protein with PYPV-Rich Domain Impairs Cuticle and Wing Development in the Asian Citrus Psyllid, *Diaphorina citri*

**DOI:** 10.3390/insects13040353

**Published:** 2022-04-02

**Authors:** Haizhong Yu, Long Yi, Zhanjun Lu

**Affiliations:** 1 College of Life Sciences, Gannan Normal University, Ganzhou 341000, China; yuhaizhong1988@163.com; 2 National Navel Orange Engineering Research Center, Gannan Normal University, Ganzhou 341000, China; 3 Ganzhou Key Laboratory of Nanling Insect Biology, Gannan Normal University, Ganzhou 341000, China

**Keywords:** *Diaphorina citri*, DcCP64, RNA interference, transcriptome sequencing

## Abstract

**Simple Summary:**

Molting is extremely important for insect growth and development, which is accompanied the degradation of old cuticle and synthesis of new cuticle. Chitin and proteins, as major components of insect cuticle, maintain the rigidity of the exoskeleton. The functions of chitin-binding proteins have not, to date, been characterized in *Diaphorina citri*. In the current study, we identified a cuticle protein (DcCP64) according to chitin column purification and LC-MS/MS analysis. Silencing of *DcCP64* induced an abnormal phenotype and increased the permeability of the abdomen and wings. Additionally, the mortality and malformation rate significantly increased, and the molting rate decreased after inhibition of *DcCP64*. Transcriptome sequencing analysis revealed that up-regulated DEGs were mainly related to oxidative phosphorylation, whereas down-regulated DEGs were mainly involved in MAPK and FoxO signaling pathways. Our results provide a basis for further functional research on *DcCP64* in *D. citri*.

**Abstract:**

Chitin is a major component of the arthropod exoskeleton, always working together with chitin-binding proteins to maintain the functions of extracellular structures. In the present study, we identified a cuticle protein 64 from *Diaphorina citri* using a chitin-binding assay. Bioinformatics analysis revealed that DcCP64 contained eight conserved PYPV motifs but lacked a Rebers–Riddiford (R–R) consensus and other chitin-binding domains. RT-qPCR analysis suggested that *DcCP64* had the highest expression level in the wing and fifth-instar nymph stage. Knockdown of *DcCP64* by RNA interference (RNAi) resulted in a malformed-wing phenotype, higher mortality and decreased molting rate. Furthermore, transcriptomics analysis revealed that 1244 differentially expressed genes (DEGs) were up-regulated and 580 DEGs were down-regulated, compared with ds*DcCP64* groups and ds*GFP* groups. KEGG enrichment analysis revealed that up-regulated DEGs were mainly related to oxidative phosphorylation, whereas down-regulated DEGs were mainly involved in the MAPK and FoxO signaling pathways. Moreover, inhibition of *DcCP64* significantly affected the cuticle surface, and increased the permeability of the abdomen and wings. Further chitin- and cellulose-binding assay confirmed the chitin-binding properties of recombinant DcCP64 in vitro. These results indicate that *DcCP64* might play an important role in the cuticle and wing development of *D. citri*.

## 1. Introduction

Citrus Huanglongbing (HLB) is one of the most destructive citrus diseases, caused by *Candidatus* Liberibacter asiaticus (*C*Las) and spread by *Diaphorina citri* [1,2]. The control of HLB can be achieved by suppressing either the bacterium or the transmission vector. The most effective method is vector control of Asian citrus psyllid (ACP) [3]. To date, the control of *D. citri* mainly relies on using chemical insecticides, including neonicotinoids, pyrethroid and organophosphate [4]. However, excessive use of chemicals has caused serious environmental damage and contributed to pest resistance [5]. Thus, there is a great need to develop new strategies for ACP management that are more specific, efficient, and sustainable.

During development, insects must shed their old exoskeleton and synthesize a new cuticle to grow and undergo metamorphosis [6]. The insect cuticle is a biological composite material consisting of chitin-fiber plies and chitin-binding proteins, which can protect its carriers against dehydration and constitutes a physical barrier to prevent various pathogens [7]. The crystalline chitin filaments are embedded in the proteinaceous matrix to form a two-layer glycoprotein complex comprising the exocuticle and endocuticle, which plays an important role in maintaining insect shape and protecting insects [8,9]. In addition, chitin-binding cuticle proteins also play crucial roles in maintaining the structure of the cuticle and numerous physiological functions. To date, several types of chitin-binding cuticle proteins have been identified and analyzed in various insects [10,11,12]. Identification of chitin-binding protein is a critical step toward increasing our understanding of the structure of insect cuticle.

Cuticular protein (CP) genes usually account for ˃ 1% of the total genes in an insect genome [13]. To date, many CP family members have been identified in various insect species, including *Bombyx mori* [13], *Spodopteta litura* [14], *Anopheles gambiae* [15], *Locusta migratoria* and *Nilaparvata lugens* [16,17]. Insect CPs are classified into several distinct families defined by the presence of specific amino acid sequence motifs, namely, CPR, CPF, CPG, CPT, and CPFL, according to the Rebers–Riddiford (R–R) consensus [18]. The CPR family, as the largest CP family which contains a conserved amino acid sequence motif known as the proteins with R–R consensus, can be split into three groups: RR-1, RR-2 and RR-3 R–R consensus [13]. The CPF family members have a characteristic stretch of 42–44 amino acid residues with C-terminal similarity among family members [15]. The CPG family members contain a conserved glycine-rich region, with a GGx (0–1) GG motif [13]. In addition to these identified cuticle proteins, it is important to note that many new and unclassified members have been added to a growing cuticular protein database. During the growth and development of *D. citri*, molting is a typical phenomenon, especially in the process of transition from nymph to adult. However, the specific functions of CPs in *D. citri* have not been reported.

RNA interference (RNAi) has been developed as an effective gene-silencing tool in animals and plants, and initiated by double-stranded RNA (dsRNA) that is homologous in sequence to the target genes [19]. In addition, RNAi has also shown great potential in pest management [20]. However, many factors limiting RNAi efficiency have been reported, such as incomplete dsRNA internalization, instability of dsRNA, impaired systemic spreading of the RNAi signal, and refractory target genes [21]. Therefore, an efficient dsRNA delivery method and selection of appropriate target genes are the two prerequisites for RNAi-mediated pest management. In *D. citri*, for different target genes, the dsRNA delivery methods need to be changed accordingly. Yuan et al. revealed that it was possible to silence *D. citri NADPH-cytochrome P450 reductase* (*DcCPR*) by RNAi using the parafilm feeding method, and the results showed that the application of dsRNA-*DcCPR* was sufficient to repress *DcCPR* expression and increase the susceptibility of *D. citri* to imidacloprid and thiamethoxam [22]. Using an artificial diet mixed with dsRNA, RNAi of *DcGSTe2* and *DcGSTd1* increased the mortalities of thiamethoxam-treated psyllid by 23% and fenpropathrin-treated psyllid by 15% [23]. In our previous research, silencing of *D. citri trehalase* (*DcTre*) gene by RNAi significantly reduced the expression levels of chitin metabolism-related genes and led to a malformed phenotype, and dsRNA delivery was conducted by an artificial diet [24].

In the present study, a cuticular protein 64, named as DcCP64, was isolated from the *D. citri* soluble proteins using chitin-binding assay in combination with LC-MS/MS analysis. To identify the molecular function of *DcCP64*, we investigated the spatial-temporal expression patterns of *DcCP64* and demonstrated the effects of silencing *DcCP64* expression on nymph survival, molting process, cuticle structures and wing development. Finally, a chitin- and cellulose-affinity assay was conducted to further confirm the chitin-binding properties of recombinant DcCP64 in vitro.

## 2. Materials and Methods

### 2.1. D. citri Rearing and Sample Collection

*D. citri* were reared using *Murraya exotica* in the College of Life Sciences, Gannan Normal University, Ganzhou, China. The rearing condition was controlled at 27 ± 1 °C, 70% ± 5% relative humidity and a 14:10 dark–light cycle. To keep the consistency of *D. citri* growth and development, the mated *D. citri* females were released into the flourishing *Murraya exotica* with many bud breaks in an insect rearing cage. After 48 h, all *D. citri* adults were removed using a portable aspirator. According to *D. citri* morphological features, each nymph was classified into its instars under a stereomicroscope, and collected using a clean soft brush. All collected samples were reserved at −80 °C. Each group of samples consisted of three biological replicates.

### 2.2. RNA Extraction, cDNA Synthesis and RT-qPCR Analysis

*D. citri* adults were dissected under a stereoscopic microscope to obtain various tissues, including head, leg, wing, integument and midgut. *D. citri* egg and nymph at different instars (first-, second-, third-, fourth-, and fifth-instar nymphs) were collected using a hair brush. All samples contained three biological replicates. The total RNA of each sample was isolated using TRIzol reagent (Invitrogen) according to the manufacturer’s instructions. The ratios of A_260/230_, A_260/280_ and the RNA concentration were assayed using a NanoDrop 2000 spectrophotometer (Thermo Fisher Scientific, New York, NY, USA.). The integrity of total RNA was confirmed using 1% agarose gel electrophoresis.

The concentration of each RNA sample was adjusted to 1 μg/μL with RNase-free water and total RNA was reverse-transcribed in a 20 μL reaction system using the PrimeScript™ RT Reagent Kit with gDNA Eraser (TaKaRa, Dalian, China). Briefly, 2.0 μL of 5 × gDNA Eraser buffer, 1.0 μL gDNA Eraser, and 1.0 μg total RNA were mixed in a PCR tube and then RNase-free water was added to 10 μL, and the solution was incubated at room temperature for 5 min. An amount of 4.0 μL 5 × PrimeScript buffer, 1.0 μL PrimeScript RT Enzyme Mix I, and 1.0 μL RT Primer Mix was added to the previous tube, then RNase-free water was added to 20 μL, and the solution was incubated at 37 °C for 15 min followed by 85 °C for 5 s, and stored at −80 °C for later use.

RT-qPCR was performed to analyze the expression levels of *DcCP64* in different tissues and developmental stages. The primers are presented in Table 1. The reaction mixture was prepared in a clean 1.5 mL centrifuge tube containing 8 μL of ddH_2_O, 10 μL of SYBR Ⅱ, 0.5 μL of forward primer, 0.5 μL of reverse primer and 1.0 μL of cDNA template. The reaction procedures consisted of an initial denaturation at 95 °C for 60 s and 40 cycles at 95 °C for 10 s, 60 °C for 10 s, and 72 °C for 10 s. The reactions were performed with a LightCycler^®^ 96 PCR Detection System (Roche, Basel, Switzerland). The relative expression levels were analyzed using the 2^−ΔΔCt^ method. The reference gene was *glyceraldehyde-3-phosphate dehydrogenase* (*GAPDH*). The primer amplification efficiency for DcCP64 and GAPDH are 97.24% and 94.13%, respectively. All experiments contained three biological replicates.

### 2.3. cDNA Library Preparation and Illumina Sequencing

The cDNA library preparation and Illumina sequencing were performed at Novogene Biological Information Technology Co., Ltd. (Tianjin, China). RNA concentration and purity were measured according to a Qubit RNA Assay Kit in a Qubit^®^ 2.0 Fluorometer (Life Technologies, Grand Island, NY, USA). In total, 1 µg RNA was used to construct the cDNA library by TruSeq RNA Sample Preparation Kit v2 (Illumina, San Diego, CA, USA) according to the manufacturer’s instructions.

The prepared cDNA library was sequenced by the Illumina HiSeq platform, and generated 150-bp paired-end reads. The clean reads were obtained by removing reads containing the adapter from the raw data. Additionally, the Q20, the Q30 and the GC-content of the clean data were calculated.

### 2.4. Read Mapping, DEGs Identification and Functional Annotation

The transcriptome data were mapped to *D. citri* reference genome (ftp://ftp.citrusgreening.org/annotation/OGSv2.0 (accessed on 12 January 2022)) using Hisat2 (version 2.0.5; https://anaconda.org/biobuilds/hisat2 (accessed on 12 January 2022)) aligner. This generated a database of splice junctions based on the gene model annotation file. The expression levels of these genes were calculated using reads per kilobase of exon per million reads mapped. Differential expression analyses of genes between ds*GFP* groups and ds*DcCP64* groups were performed using the DESeq2 R package. *p*-values were adjusted using the Benjamini–Hochberg method to control for the false-discovery rate. A corrected *p*-value of 0.05 and an absolute |log2 (fold change)| (Fold change > 1) of 0 were set as the thresholds for significantly differential gene expression. The hierarchical cluster analysis of DEGs was conducted using Genesis software (http://genome.tugraz.at/genesisclient_download.shtml (accessed on 12 January 2022)).

Gene ontology (GO) is a tool used for gene annotation by collecting a defined, structured and controlled vocabulary. The topGO R package, which implements the GO terms, was used for the enrichment analysis of length-corrected DEGs. Kyoto Encyclopedia of Genes and Genomes (KEGG) is a database that can be used to understand the high-level functions and utilities of biological systems, such as cells, organisms and ecosystems from a molecular level. A KEGG pathway enrichment analysis for DEGs was performed using KOBAS. A *p*-value of < 0.01 was set as the threshold. 

### 2.5. D. citri Total Proteins Isolation

*D. citri* total proteins were extracted from the adults of *C*Las-free ACPs according to the previously described protocols with some modifications [25]. In brief, a total of 1000 *D. citri* adults were ground into powder using liquid nitrogen, and the sample was homogenized in 1 mL of pre-cooled PBS buffer (pH 7.4). The homogenate was centrifuged at 12,000× *g* for 10 min at 4 °C to separate the supernatant and sediment. The supernatant (PBS-1) was transferred to a new tube for further analysis. The above procedure was repeated to obtain the supernatant (PBS-2). The pellet was collected, and resuspended into 1 mL 2% SDS crude extract buffer. The mixed solution was incubated at 12 °C for 1 h, and centrifuged at 12,000× *g* for 20 min at 4 °C to collect the supernatant (2% SDS-1). The same extraction procedure was repeated twice to obtain 2% SDS-2 and 2% SDS-3. Most of the SDS was removed from the above protein solutions by adding 1 M KCl solution to cause precipitation of the potassium salt. The protein supernatant was recovered by centrifugation at 12,000× *g* for 20 min at 4 °C, and then was diluted 20-fold by 1 mM HEPES (pH 7.4). To completely remove the residual SDS, the diluted protein solutions were concentrated at 12,000 g, 4 °C for 10 min. The protein concentrations were quantified using a BCA Protein Assay Kit (Bio-Rad, Hercules, CA, USA).

### 2.6. Chitin and Cellulose Binding Properties Analysis

The chitin- and cellulose-binding properties were analyzed according to previous protocol with some modifications [26]. In brief, 1 mL of chitin beads (New England BioLabs S6651V, Beverly, MA, USA) and 2 g of cellulose powder (Sigma-Aldrich C6288-100G, St Louis, MO, USA) were added to the balance column (GenScript, Nanjing, China), and then 5 mL binding buffer (20 mM HEPES and 500 mM NaCl, pH 7.4) was added. The mixture was equilibrated at room temperature for 2 h and kept at 4 °C until use. One milliliter of *D. citri* proteins or DcCP64 recombinant protein was incubated with 3 mL of the prepared chitin or cellulose mixture for 4 h at room temperature. Then, the mixed solution was added to the balance column and washed using 5 mL washing buffer (20 mM HEPES, pH 7.4 and 1 M NaCl). This step was repeated three times and the washing liquid was collected. Finally, the proteins bound to the beads were eluted with 8 M of urea, and the concentration of protein was determined using a BCA Protein Assay Kit (Sangon Biotech, Shanghai, China) according to the manufacturer’s instructions. All samples were analyzed by 10% SDS-PAGE and visualized by staining with Coomassie brilliant blue (CBB).

### 2.7. In-Gel Digestion and LC-MS/MS Analysis

The target bands were cut and washed twice using sterile water, and then de-stained in 50% acetonitrile containing 50 mM NH_4_HCO_3_. The decolorized gel pieces were further dehydrated with 100% acetonitrile for 5 min. The gel pieces were then rehydrated with 10 mM dithiothreitol and were incubated at 56 °C for 1 h. After double dehydration of the gel pieces in 100% acetonitrile, the remaining liquid was removed and the gel pieces were rehydrated in 55 mM iodoacetamide. All samples were placed at room temperature for 45 min under dark conditions. The gel pieces were washed with 50 mM NH_4_HCO_3_ on ice for 1 h. Excess liquid was removed and the gel pieces were digested with trypsin at 37 °C overnight. Peptides were extracted with 50% acetonitrile, followed by 100% acetonitrile. Peptides were dried and resuspended in 0.1% formic acid.

The peptides were dissolved in 0.1% formic acid, and loaded onto a reversed-phase analytical column (15 cm length, 75 cm i.d.). The gradient increased from 6% to 23% (0.1% formic acid in 98% acetonitrile) over 16 min, 23% to 35% in 8 min, climbing to 80% in 3 min, then holding at 80% for 3 min, all at a constant flow rate of 400 nL/min, as measured on an EASY-nLC 1000 UPLC system. The peptides were subjected to an NSI source followed by tandem mass spectrometry (MS/MS) in a Q ExactiveTM Plus (Thermo Fisher Scientific) coupled online to the UPLC. The applied electrospray voltage was 2.0 kV. The m/z scan range was 350 to 1800, for full scan. The intact peptides were detected in the Qrbitrap at a resolution of 70,000. Peptides were then selected for MS/MS using an NCE setting of 28, and the fragments were detected in the Qrbitrap at a resolution of 17,500. A data-dependent procedure alternated between one MS scan followed by 20 MS/MS scans with 15.0 s dynamic exclusion. Automatic gain control (AGC) was set at 5E4.

### 2.8. Protein Identification and Bioinformatics Analysis

The obtained MS/MS raw data were analyzed using Proteome Discoverer 1.3 software. All tandem mass spectra were searched against the *D. citri* protein database (https://www.citrusgreening.org/ (accessed on 12 January 2022)). Mass error was set to 7 ppm for precursor ions and 0.02 Da for fragment ions, and a peptide ion score of 20 was set as a threshold. The cDNA and deduced amino acid sequence of DcCP64 were analyzed using DNASTAR software and online BLAST software (http://www.ncbi.nlm.nih.gov/blast (accessed on 12 January 2022)). The signal peptide sequence of *DcCP64* was predicted with the SignalP4.1 server (http://www.cbs.dtu.dk/services/SignalP/ (accessed on 12 January 2022)). The molecular weight and isoelectric point of DcCP64 were calculated by using an online tool at http://web.expasy.org/compute_pi (accessed on 12 January 2022). The structural domain was analyzed by using the SMART database (http://smart.embl-heidelberg.de/ (accessed on 12 January 2022)). A phylogenetic tree was constructed by the neighbor-joining method with 1000-fold bootstrap resampling using MEGA 5.0 software (Center for Evolutionary Medicine and Informatics, Tempe, AZ, USA).

### 2.9. RNAi-Mediadted DcCP64 Silencing

According to the sequence of *DcCP64*, the specific primers with T7 promoters were designed, and are presented in Table 1. The ds*DcCP64* and de*GFP* were synthesized using the T7 RioMAX Express RNAi System (Promega, San Luis Obispo, CA, USA) following the manufacturer’s instructions. The soaking delivery of dsRNA was performed according to a previous report [27]. Briefly, the concentration of synthetic dsRNA was measured using a NanoDrop 2000 spectrophotometer (Thermo Fisher Scientific) and was diluted to 500 ng/µL using DEPC-treated water containing 15% sucrose and 0.1% blue food dye. A total of 150 were soaked in dsRNA solution for 5 min and then transferred onto the fresh M. exotica seedlings. All experiments contained three biological replicates. To prevent *D. citri* nymphs escaping and to allow ventilation, the upper openings of the cages were covered with an insect-proof mesh screen. All living *D. citri* were collected at 24 h and 48 h after dsRNA treatment. The effects of ds*DcCP64* on gene expression were analyzed using RT-qPCR.

### 2.10. Eosin Y Staining and Scanning Election Microscopy (SEM) Analysis

Eosin Y staining was performed based on previous protocol [28]. Briefly, *D. citri* after dsRNA treatment were collected and anaesthetized with CO_2_, and incubated in 1 mL dye solution containing 0.5% Eosin Y (W/V) and 0.1% Triton X-100 at 55 °C for 1 h. Stained *D. citri* were washed three times with distilled water, and mounted onto glass slides. Images were collected using a MV PLAPO 1× microscope (Olympus America Inc., Melville, NY, USA).

The wing and cuticle structures after silencing of *DcCP64* were examined using a scanning electron microscope (SEM). For SEM analysis, the adult wing and cuticle were dissected and fixed in 2.5% glutaraldehyde (pH 7.0) for 2 h at 4 °C, and then fixed with 1% osmic acid at 4 °C for 3 h. After fixation, all samples were dehydrated in ethanol (50%, 70%, 80%, 85%, 90%, 95%, and 100%) for 15 min at 4 °C. The dehydrated samples were consecutively soaked by penetrant 1 (2:1 mixture of acetone and epoxy resin), penetrant 2 (1:1 mixture of acetone and epoxy resin), and penetrant 3 (epoxy resin) at 37 °C for 12 h. All samples were dried with liquid CO_2_ at the critical point and coated with platinum with a sputter coater at 4 mA for 3 min. Finally, the samples were observed with S-4800N SEM (Hitachi, Tokyo, Japan).

### 2.11. Protein Expression and Purification

Based on the sequence of *DcCP64*, we designed specific primers containing restriction enzyme sites to apply the open reading sequence (Table 1). The purified PCR product was cloned into pMD19-T (Novagen, WI, USA), and ligated into the pET-28a vector (Novagen). The recombinant vector was subjected to DNA sequencing, and transformed into BL21 (DE3) (TaKaRa, Dalian, China) competent cells for protein expression. Different concentrations of isopropyl β-D-thiogalactoside (IPTG) were added to optimize protein expression. The recombinant protein was induced with 0.6 mM IPTG at 37 °C overnight. The cells were harvested and resuspended in binding buffer (20 mM Tris-HCl, 500 mM NaCl, and 5 mM imidazole, pH 7.9). The mixture was disrupted by sonication on ice, and purified using Ni-NTA Fast Start Kit (GenScript, Nanjing, China) following the manufacturer’s instructions. The purified protein was analyzed by 12% sodium dodecyl sulfate-polyacrylamide gel electrophoresis (SDS-PAGE) and Western blotting.

Western blot was performed according to previous protocol. Briefly, the protein samples were separated by 12% SDS-PAGE and transferred onto polyvinylidene difluoride (PVDF) membrane. The membranes were blocked using 5% non-fat milk in PBST consisting of 137 mM NaCl, 2.7 mM KCl, 10 mM Na_2_HPO_4_, 2 mM K_2_HPO_4_, and 0.1% (*v*/*v* Tween-20) for 1 h at room temperature, washed with PBST, and then incubated with 1:10,000 anti-His primary antibody (Transgen Biotech, Beijing, China) for 3 h at room temperature. After washing, antigen antibody complexes were detected using a 1:5000 horseradish peroxidase-conjugated goat anti-mouse secondary antibody (Transgen Biotech) in blocking buffer for 1 h. After washing three times, immobilized conjugates on the membrane were visualized in horseradish peroxidase substrate solution (Tiangen, Beijing, China).

## 3. Results

### 3.1. Identification of the DcCP64 Gene and Bioinformatics Analysis

**Figure 1:**
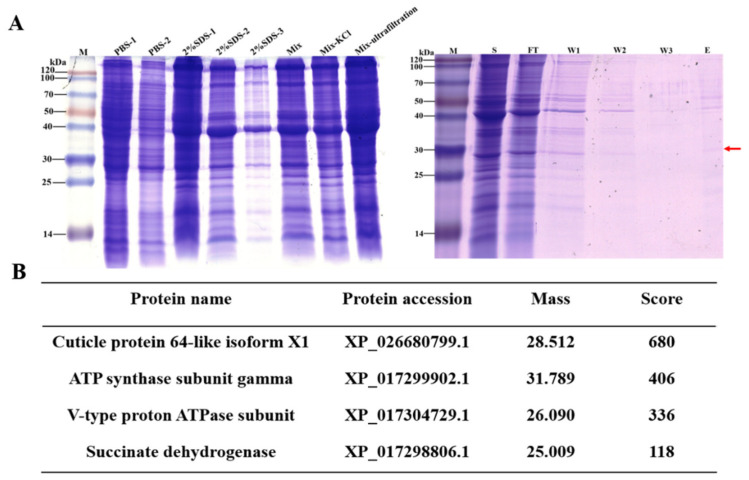
Extraction of *D. citri* cuticle protein 64 (DcCP64) by chitin-binding experiment combined with LC-MS/MS analysis. (**A**) SDS-PAGE analysis of proteins from *D. citri* adults, and chitin-binding assays using the mix ultrafiltration. Mix indicated the mixture of proteins isolated by PBS and 2% SDS; (**B**) Identification of DcCP64 by LC-MS/MS analysis.

**Figure 2:**
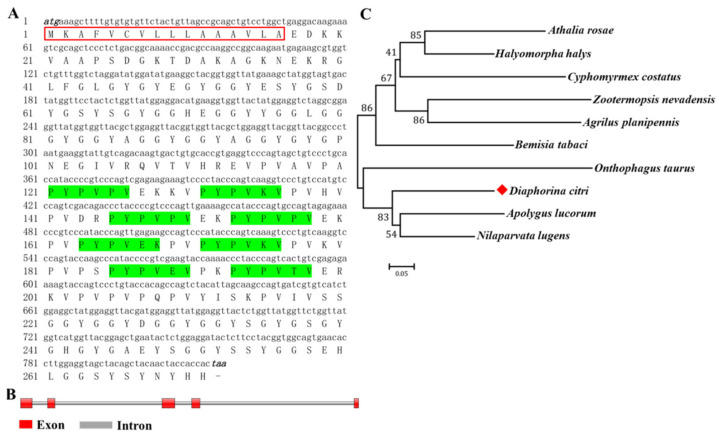
Bioinformatics analysis of DcCP64. (**A**) Complete nucleotide and deduced amino acid sequence of *D. citri CP64* (*DcCP64*) cDNA. Numbers on the left side represent nucleotide and amino acid positions. The initiation codon (ATG) and termination codon (TAA) are indicated in black italics. The signal peptide sequence is indicated by a red box. Eight PYPV motifs are marked by a green region. (**B**) Gene structure analysis of *DcCP64* by using Splign online software. (**C**) Phylogenetic relationships of DcP64 in different insect species using the neighbor-joining method with a bootstrap value of 1000. The protein accessions are as follows: *Diaphorina citri* (XP_008474070.1), *Apolygus lucorum* (KAF6213364.1), *Bemisia tabaci* (XP_018909777.1), *Zootermopsis nevadensis* (XP_021934705.1), *Onthophagus taurus* (XP_022901515.1), *Agrilus planipennis* (XP_018319890.1), *Athalia rosae* (XP_012251808.1), *Nilaparvata lugens* (XP_039293390.1), *Halyomorpha halys* (XP_014277355.1), and *Cyphomyrmex costatus* (XP_018400946.1). The red rhombus mark indicated *Diaphorina citri*.

*D. citri* total protein was isolated by PBS and 2% SDS, respectively. The results showed that ultrafiltered proteins were found with a molecular weight ranging from 14 kDa to 100 kDa (Figure 1B). The obtained supernatants were incubated with chitin beads, and further washed by washing buffer. Eventually, several protein bands were detected by SDS-PAGE analysis, and were submitted to LC-MS/MS analysis (Figure 1A). The *DcCP64* protein (GenBank Accession No: XM_008475848.3) was identified from LC-MS/MS analysis (Figure 1B). The cDNA sequence of *DcCP64* contained an ORF of 816 bp encoding a protein of 271 amino acid residues with a predicted MW of 28.5 kDa and pI of 8.24 (Figure 2A). The deduced protein DcCP64 was speculated to be a secreted protein with a signal peptide of 16 amino acids in the N-terminal region. In addition, eight conserved motifs (PYPVxx) were identified (Figure 2A). Gene structure analysis showed that *DcCP64* contains of five exons and four introns (Figure 2B). Based on the amino acid sequences of CP64 from different insect species, a phylogenetic tree was constructed using MEGA 5.0 to investigate the evolutionary relationship of DcCP64. The results showed that DcCP64 had a close relationship with the sap-sucking hemiptera insects, including *Apolygus lucorum* and *Nilaparvata lugens* (Figure 2C).

### 3.2. Expression Patterns of DcCP64 in Different Tissues and Developmental Stages

**Figure 3:**
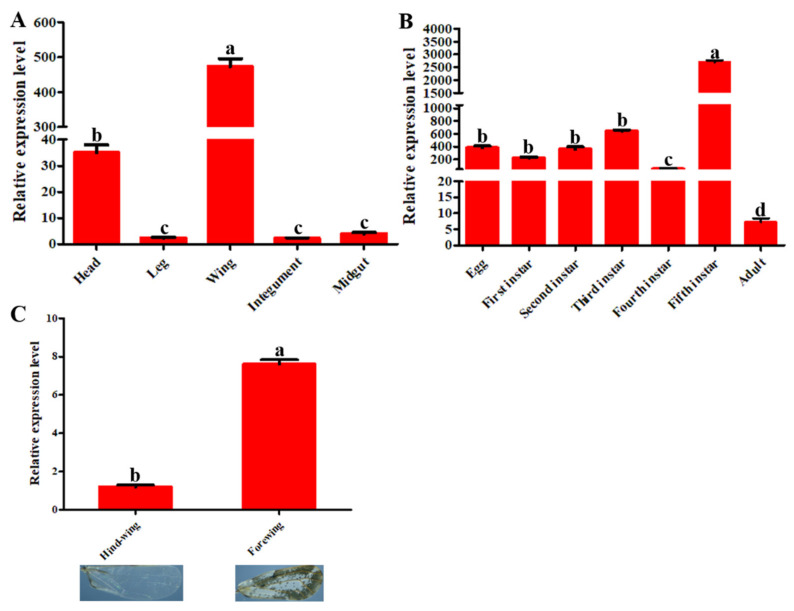
Expression patterns of *DcCP64* in different tissues, different developmental stages and fore/hind-wing of *D. citri*. Relative expression level of *DcCP64* was analyzed using RT-qPCR. (**A**) Relative expression levels of *DcCP64* in different tissues of *D. citri*. (**B**) Relative expression levels of *DcCP64* in different developmental stages of *D. citri*. (**C**) Relative expression levels of *DcCP64* in fore-wing and hind-wing of *D. citri*. Datas were normalized using *glyceraldehyde-3-phosphate dehydrogenase* (*GAPDH*) and are represented as the means ± standard errors of the means from three independent experiments. Relative expression levels were calculated using the 2^−ΔΔCt^ method. Statistical analysis was conducted using SPSS software. Significant differences are indicated by different letters, for example, (**a**), (**b**), (**c**) and (**d**) (*p* < 0.05).

The expression patterns of *DcCP64* in different tissues and different developmental stages were investigated by RT-qPCR. The results showed that the *DcCP64* gene was expressed in all tissues, including head, leg, wing, cuticle and midgut. A higher expression level of *DcCP64* was detected in the wing and head tissues. The expression levels of *DcCP64* in the wing and head were 206.0 and 15.2 times more than in the integument, respectively. In addition, the expression level of *DcCP64* in the forewing was significantly higher than in the hindwing (Figure 3). The expression level of *DcCP64* was constantly observed without significant differences from egg to third-instar nymph stage, with a significant decrease at the fourth-instar nymph stage (Figure 3). The peak expression level was observed at the fifth-instar stage, while the lowest expression level occurred at adult stage (Figure 3). The expression level of *DcCP64* in the fifth-instar nymph was 375.1 times that of the adult.

### 3.3. RNAi-Based Silencing of DcCP64 and Phenotypic Analysis

**Figure 4:**
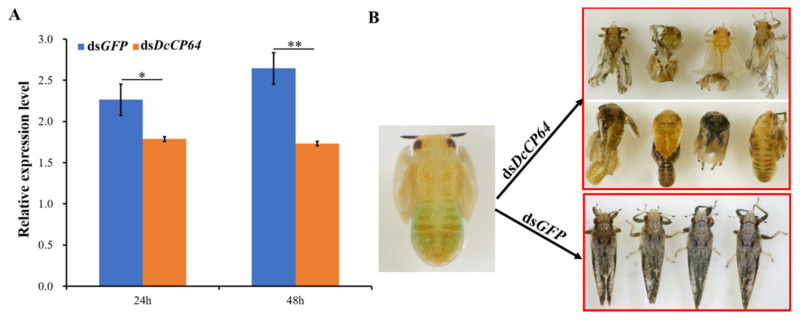
Effects on *D. citri* after RNA interference (RNAi) of *DcCP64*. (**A**) Relative expression levels of *DcCP64* when *D. citri* was treated with ds*DcCP64* and ds*GFP*. The mean expression level represented for three biological replicates. The relative expression levels were calculated using the 2^−ΔΔCt^ method. Statistical analysis was performed using SPSS software. The significant differences are indicated by * *p* < 0.05 and ** *p* < 0.01. (**B**) Representative phenotypes of *D. citri* at 48 h post RNAi treatment.

To determine the effect of *DcCP64* on *D. citri* molting, RNAi was performed by soaking of dsRNA. At 24 h and 48 h after treatment with ds*DcCP64*, the expression level of *DcCP64* was significantly down-regulated compared with the controls (ds*GFP*) (Figure 4A). In the ds*DcCP64* treatment group, the transition from fifth-instar nymph to adult was disrupted, and the emerged *D. citri* adult had two different phenotypes. The first phenotype was that the treated fifth-instar nymphs molted into adults with abnormal dorsal tergites and/or malformed wings. Wings in these adults were irregular in shape, curled at the distal end or smaller in size. The other phenotype was that the fifth-instar nymphs failed to completely molt. However, in the ds*GFP* control group, the fifth-instar adult could undergo a normal molt (Figure 4B).

**Figure 5:**
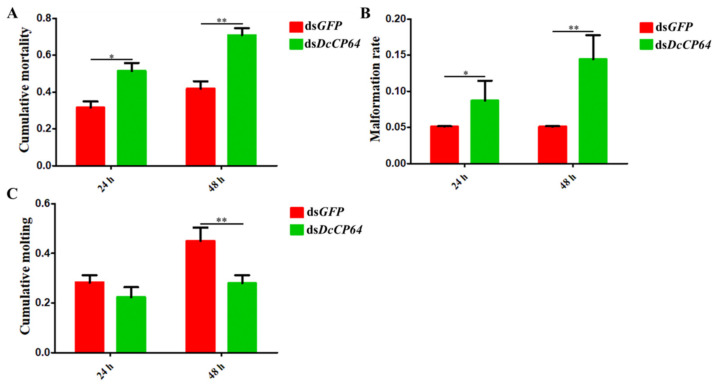
Effects of RNAi on *D. citri* mortality, malformation and molting. (**A**) Detection of cumulative mortality of *D. citri* at 24 h and 48 h after RNAi of *DcCP64*. The ds*GFP* treatment group was used as a control. (**B**) Detection of malformation rate of *D. citri* at 24 h and 48 h after RNAi of *DcCP64*. (**C**) Detection of cumulative molting of *D. citri* at 24 h and 48 h after RNAi of *DcCP64*. The significant differences are indicated by * *p* < 0.05 and ** *p* < 0.01. Statistical analysis was performed using SPSS software.

Importantly, the cumulative mortality and malformation rates significantly increased after silencing of the *DcCP64* gene at 24 h and 48 h. The cumulative mortality in the ds*DcCP64* treatment group was 50% compared with 28% in the control group at 24 hpt, and the cumulative mortality in the treatment group reached 72% at 48 hpt (Figure 5A). The malformation rate in the ds*GFP* control group was 5%, and had no significant change from 24 hpt to 48 hpt, while the malformation rate in the ds*DcCP64* treatment group increased from 8% to 15% during this period (Figure 5B). On the contrary, for the rate of the cumulative molting between 24 hpt and 48 hpt, no significant difference was observed on the ds*DcCP64*-treated *D. citri*, while it increased from 27% to 42% in the control group (Figure 5C). These results indicated that silencing of *DcCP64* impaired the molting process of fifth-instar nymphs.

### 3.4. Permeability and Microstructure Analysis after Silencing of DcCP64

**Figure 6:**
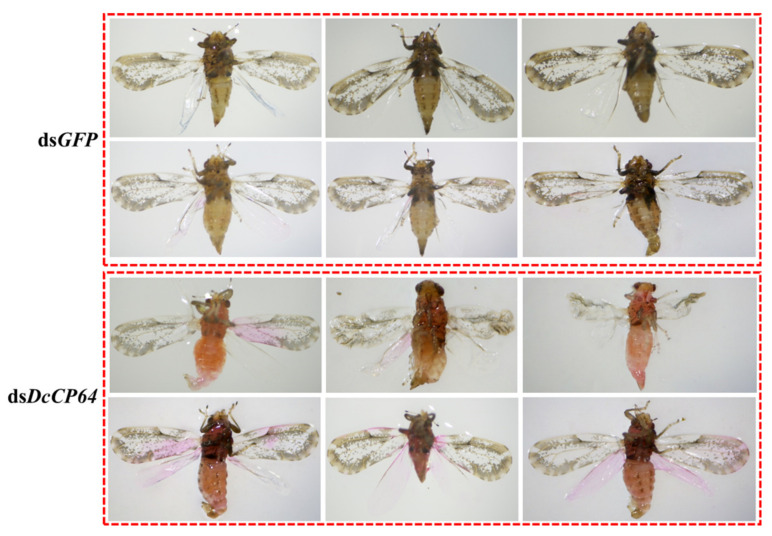
Effects of silencing of *DcCP64* on wing and cuticle permeability. Silencing of *DcCP64* caused excessive Eosin Y penetration in the forewing and entire cuticle.

To assess cuticle permeability after silencing of *DcCP64*, we conducted Eosin Y penetration tests at 55 °C. The results showed that the wing and abdominal cuticle were obviously stained by Eosin Y in the dsDcCP64 group, and the pink color that originated from Eosin-Y was mainly distributed in the wing root (Figure 6). By contrast, no pink color was detected in the wing and cuticle of the control. These results indicated that silencing of DcCP64 enhanced the permeability of the wing and cuticle.

**Figure 7:**
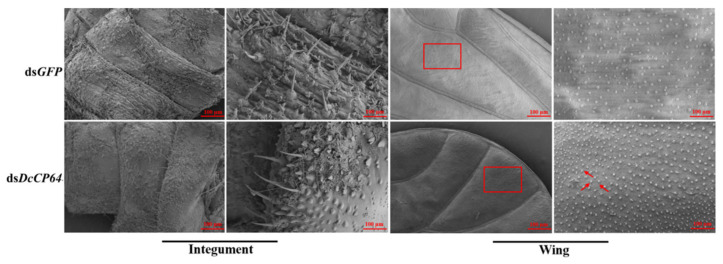
Microstructural analysis of *D. citri* wing and cuticle after RNAi of *DcCP64* by SEM. The red box indicates the differences in surface structure. The red arrows indicate the spiny trunk on *D. citri* surface of the wing.

We speculated that silencing of *DcCP64* might change the surface structure of the wing and cuticle, thereby enhancing their permeability. To test this conjecture, SEM was performed at 48 h after RNAi treatment. The results showed that the surface of the wing in the ds*DcCP64* treatment group was rough, while the surface of control *D. citri* was relatively smooth. Notably, a rough integument surface was also observed in the RNAi psyllids. In contrast, ds*DcCP64* treatment did not alter the structure of the integument surface in *D. citri* (Figure 7). These results indicated that *DcCP64* might be involved in the process of maintaining permeability of the wing and cuticle.

### 3.5. Verify the Chitin-Binding Using Recombinant DcCP64 Protein

**Figure 8:**
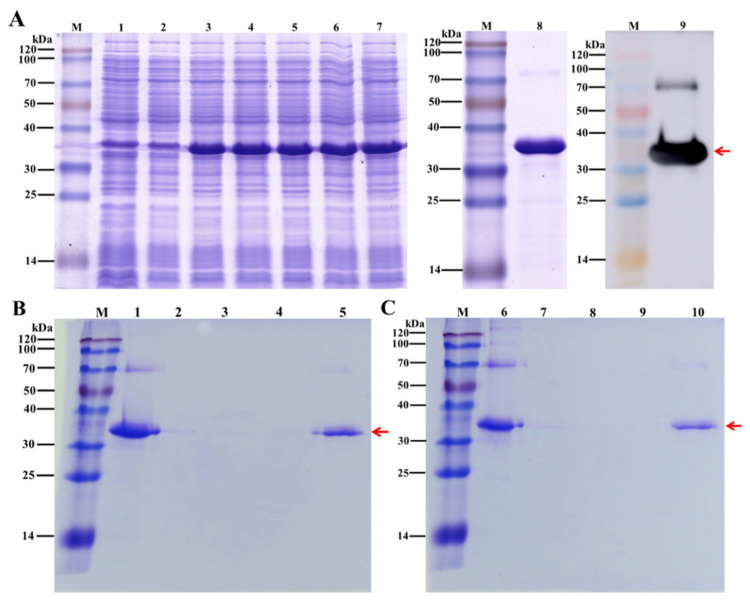
Analysis of the recombinant DcCP64 protein using SDS-PAGE and binding assays of recombinant protein DcCP64 to chitin and cellulose. The cellulose-binding assay was used as a control. (**A**) M: molecular weight markers. Lane 1: blank control without insert. Lane 2: negative control without induction. Lanes 3–7: induced expression under IPTG concentrations of 0.2, 0.4, 0.6, 0.8 and 1.0 mmol/L, respectively. Lane 8: purified recombinant DcCP64 protein. Lane 9: Western blotting analysis of recombinant His-tagged DcCP64 protein identified by anti-His antibodies. (**B**) and (**C**) Binding assays of recombinant protein DcCP64 to chitin and cellulose. Lane1 and Lane 6: flow-through. Lane (2–4) and Lane (7–9): the first and third washing fraction. Lane 5 and Lane 10: proteins eluted by 8 M urea. The red arrow indicates the recombinant DcCP64 protein.

The recombinant DcCP64 protein was expressed using a prokaryotic expression system, and its chitin-binding property was analyzed. The recombinant DcCP64 protein, represented by a band of approximately 32 kDa, was detected by SDS-PAGE (Figure 8A). A consensus protein band was confirmed by a Western blot analysis using an anti-His antibody (Figure 8A). To verify the chitin-binding properties of DcCP64, an in vitro assay was performed using chitin- and cellulose-affinity chromatography, followed by SDS-PAGE analysis. As shown in Figure 8B,C, DcCP64 could tightly bind to colloidal chitin and cellulose.

### 3.6. Transcriptome Sequence and Assembly

After removing the redundant and short reads, 44,868,146 (97.6%), 43,994,220 (98.2%), and 45,576,636 (94.1%) clean reads from the treatment groups (ds*DcCP64*); and 43,320,742 (98.6%), 42,224,822 (98.6%), and 42,994,992 (98.3%) clean reads from the control groups (ds*GFP*) were obtained. The average Q20 (sequencing error rate < 1%) and Q30 (sequencing error rate < 0.1%) values were greater than 96% and 90%, respectively. The GC content among the different samples was approximately 40% (Table S1). In addition, 35,928,557 (80.1%), 35,581,714 (80.9%) and 35,751,775 (78.4%) clean reads from the treatment groups (dsDcCP64); and 34,495,671 (79.6%), 33,719,906 (79.9%) and 34,603,244 (80.5%) clean reads from control groups (ds*GFP*) were successfully mapped to the *D. citri* genome (Table S2). The accuracy of the sequencing data was sufficient for further analyses.

### 3.7. Identification of DEGs and Functional Annotation

**Figure 9:**
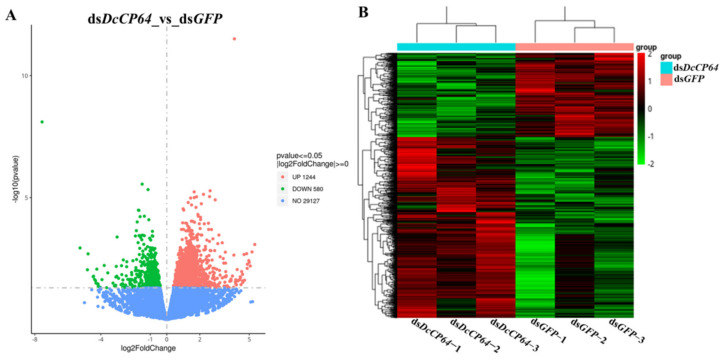
The identification and hierarchical cluster analysis of differentially expressed genes (DEGs) in treatment groups (ds*DcCP64*) and control groups (ds*GFP*). (**A**) A scatter diagram for each gene. The blue, red and green points represent no difference in expression, up-regulated genes and down-regulated genes, respectively. (**B**) Hierarchical clustering of DEGs between ds*DcCP64* and ds*GFP* groups. Columns indicate different samples. Rows represent different DEGs. Green bands indicate a low expression level, and red bands indicate a high gene expression level.

In order to use the DESeq method, DEGs were identified between control groups (ds*GFP*) and treatment groups (ds*DcCP64*). In total, 1824 DEGs were identified in ds*DcCP64* groups compared with ds*GFP* groups, among which 1244 DEGs were up-regulated, and 580 DEGs were down-regulated (Figure 9A; Table S3). Based on the log10^(RPKM+1)^ values of the three groups, the hierarchical clustering of the DEGs was performed to determine the expression patterns of the identified genes (Figure 9B).

**Figure 10:**
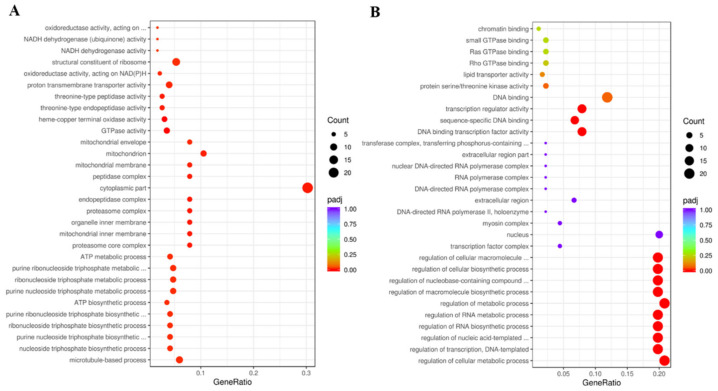
Gene ontology (GO) enrichment analysis of DEGs. A scatter diagram of GO categories. The *x*-axis indicates the gene ratio. The *y*-axis indicates different categories. (**A**) Up-regulated DEGs. (**B**) Down-regulated DEGs.

**Figure 11:**
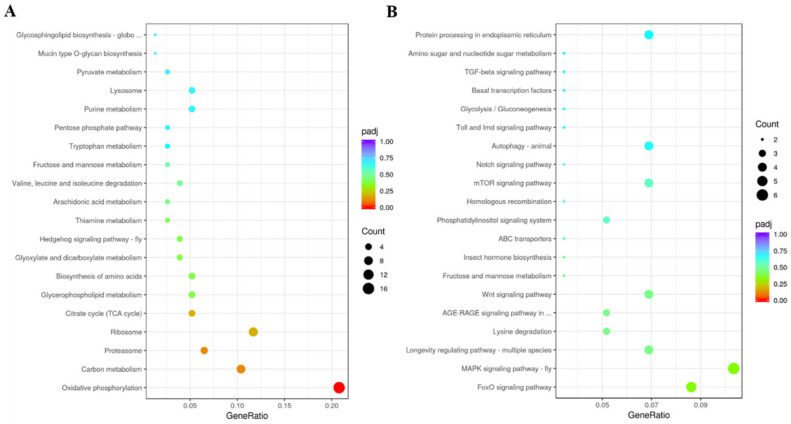
KEGG (Kyoto Encyclopedia of Genes and Genomes) enrichment analysis of DEGs. A scatter diagram of KEGG pathways. The *x*-axis indicates the gene ratio. The *y*-axis indicates different pathways. (**A**) Up-regulated DEGs. (**B**) Down-regulated DEGs.

GO enrichment analyses were conducted to confirm the functions of the DEGs. In the ds*DcCP64*_vs_ds*GFP* groups, DEGs of up-regulation were mainly attributed to cytoplasmic part and structural constituent of ribosome, and DEGs of down-regulation were involved in regulation of metabolic process and DNA binding (Figure 10; Table S4). KEGG enrichment revealed that up-regulated DEGs were associated with oxidative phosphorylation, and down-regulated DEGs were involved in MAPK and FoxO signaling pathways (Figure 11; Table S5). 

## 4. Discussion

Insects are responsible for substantial crop losses worldwide through direct damage and transmission of plant disease, and broad-spectrum chemical insecticides will facilitate the sustainable intensification of food production [22]. However, intense use of pesticides has also caused a series of problems, such as environmental pollution, food safety and insect resistance to insecticides [28]. Among them, insecticide resistance has become an exasperating problem. In insects, cuticle is mainly made of chitin filaments embedded in cuticular proteins, and plays an important role in defending against pathogen infection and reducing insecticide penetration [29,30]. Penetration resistance refers to modification in the cuticle that will eventually slow down the penetration of insecticide molecules within the insect’s body [31]. Correlation of insecticide resistance with reduced insecticide penetration through the cuticle has been reported in many insect species, including Anopheles gambiae, *Helicoverpa armigera*, and *Myzys persicae* [32,33]. In this study, *D. citri* cuticle protein 64 (DcCP64) was identified according to a chitin-binding assay followed by LC-MS/MS analysis. The results showed that a distinct band was exhibited in eluent, but there was no corresponding band in washing buffer, indicating DcCP64 might belong to a chitin-binding protein. Bioinformatics analysis showed that DcCP64 contained a signal peptide and eight PYPV conserved regions, but it had no typical chitin-binding regions. The most abundant family of cuticle proteins contains the Rebers–Riddiford consensus (R–R consensus), which has been shown to bind chitin [34]. Other families of cuticle proteins have been reported, including CPF, CPT, CPLCA, CPLCG, CPLCW, CPCFC and CPG [14]. Lu et al. (2018) identified a cuticle protein gene 21.92 (*CP21.92*) from *Nilaparvata lugens* transcriptome database, and sequence analysis showed that *NlCP21.92* contained AAPA/V motifs but lacked an R–R consensus [35]. These results indicated that DcCP64 might belong to a new novel cuticle protein.

The relative expression level of *DcCP64* was determined in different tissues and different developmental stages. The results showed that *DcCP64* had a relatively higher expression in the wing, followed by the head, but it had low expression in the leg, integument and midgut. We speculated that *DcCP64* might be assigned to the wing-specific cuticle protein. Zhao et al. (2019) identified a wing-specific cuticular protein LmACP7 from *Locusta migratoria*, which was initially produced in epidermal cells and subsequently migrated to the exocuticle at the pre-ecdysial stage in adult wings [36]. In *Bombyx mori*, *BmWCP4* was mainly expressed in the wing disc tissues containing wing bud and trachea blast during day 2 of wandering stage [12]. In addition, we also found that *DcCP64* showed a high expression in the head. In previous research, Zhang et al. (2017) also revealed that a *B. mori cuticle protein CPAP3-G* (*BmCPAP3-G*) gene was expressed highly in the head and cuticle [37]. Insects possess a cuticle that covers all tissues exposed to the outside world including the body, the fore- and hindgut and the luminal side of the tracheae [7]. Therefore, we speculated that *DcCP64* might play an important role in maintaining the rigid structure of *D. citri* wing and head. At different developmental stages, *DcCP64* had a higher expression in the fifth-instar nymph stage. The fifth-instar nymph stage is a critical period that involves progressing from nymph stage into adult stage [38]. In the process of the *D. citri* molting period, high levels of *DcCP64* tightly attached to chitin might be necessary to maintain the rigid structure of new cuticle in *D. citri*.

The exoskeleton of insects (cuticle) is an assembly of chitin and cuticle proteins [39]. Both inhibition of cuticle protein genes expression and inhibition of chitin synthesis could affect the growth and development of insects [40,41]. RNAi has already proven its usefulness in functional genomics research on insects, but it also has considerable potential for the control of pest insects [42]. Efficient dsRNA delivery and suitable RNAi targets are the two prerequisites for RNAi-mediated insect management. In this study, we knocked down the *DcCP64* gene using RNAi. The results showed that *DcCP64* was silenced effectively at 24 h and 48 h after ingestion of dsRNA. Additionally, we also found that silencing of *DcCP64* significantly increased the cumulative mortality and malformation rate, and decreased the cumulative molting rate. In the RNAi-treated group, *D. citri* molting exhibited two different phenotypes: the first phenotype was that fifth-instar nymphs could molt, but their wings were malformed; and the other phenotype was that the fifth-instar nymphs could not completely molt. In our previous research, the RNAi of *D. citri cuticle protein 7* (*DcCP7*) gene significantly affected *D. citri* molting. Interestingly, abnormal molting was usually attributed to the case that the old cuticle failed to split at the *D. citri* head, thus twisting the wings of emerged adults [27]. In insects, chitin filaments are embedded in the proteinaceous matrix to form a two-layer glycoprotein complex comprising the exocuticle and endocuticle, which form the procuticle [43]. A comparison of the cuticle proteins from various insect species indicated that most cuticle proteins possess consensus chitin-binding domains. In the current study, *DcCP64* encoded a protein containing eight conserved PYPV repeat motifs but lacking the typical chitin binding domain of CPR proteins. Recombinant DcCP64 displayed strong chitin binding properties in vitro. We speculated that the ability of DcCP64 to bind with chitin might be associated with these PYPV repeat motifs. In addition, the deformation of the surface of *DcCP64*-depleted wings correlated with the higher sensitivity to Eosin Y penetration. In previous research, Dong et al. (2020) demonstrated that suppression of *Cht10* affected the *Drosophila melanogaster* cuticle surface, and cuticle inward permeability was also enhanced [28]. Therefore, we inferred that *DcCP64* might be involved in the formation of the inward barrier in the *D. citri* wing cuticle.

Interestingly, transcriptome analysis showed that DEGs involved in MAPK and FoxO signaling pathways were down-regulated after knockdown of *DcCP64*. MAPKs are part of well-conserved eukaryotic signaling cascades which regulate numerous cellular responses [44]. In insects, the MAPK pathway plays an important role in various biological processes, including apoptosis, cell differentiation and external stress response [45]. Multiple studies have revealed that key genes in the MAPK pathway could regulate insect cuticle penetration. Jin et al. found that the *Metarhizium acridum MAPK* gene mutant failed to penetrate the cuticle outwards on the locust cadaver [46]. In addition, MAPK was also regulated by transcription factor involved in cuticle penetration [47]. In this study, we found that silencing of *DcCP64* enhanced the permeability of the wing and cuticle. Therefore, we speculated that the MAPK pathway might regulate the expression of *DcCP64*, thus affecting *D. citri* cuticle permeability. Previous research has revealed that the insulin-PI3K-Akt-FOXO signaling pathway played a crucial role in the manipulation of wing size in migratory insects [48]. In *N. lugens*, the transcription factor FoxO could mediate wing polyphenism [49]. The tissue expression pattern showed that *DcCP64* had a high expression in the wing. Interestingly, we considered that the FoxO signaling pathway might regulate *DcCP64* to promote *D. citri* wing development.

## 5. Conclusions

In summary, we identified a chitin-binding protein (DcCP64) from *D. citri*. Spatiotemporal expression analysis revealed that *DcCP64* was highly expressed in the wing and fifth-instar nymph stage. RNAi-based gene silencing inhibited the expression of *DcCP64* and influenced the structure of wing, resulting in malformed phenotypes. Furthermore, inhibition of *DcCP64* significantly affected the cuticle surface, and increased the permeability of the abdomen and wings. The recombinant DcCP64 protein was expressed using prokaryotic expression system and exhibited obvious chitin-binding properties. KEGG enrichment analysis revealed that up-regulated DEGs were mainly related to oxidative phosphorylation, whereas down-regulated DEGs were mainly involved in MAPK and FoxO signaling pathways. Taken together, our results indicated that *DcCP64* could be used as a new target for the control of *D. citri*.

## Figures and Tables

**Table 1:** Primers used in this study.

Genes	Primer Sequence (5′-3′)	Application
*DcCP64*	F1: GCATTCAATCATCAAACCGAG	RT-qPCR analysis
	R1: AGACCAAACAGACCACGCTTC	
*GAPDH*	F2: CATGGCAAGTTCAACGGTGA	
	R2: CGATGCCTTCTCAATGGTGG	
*DcCP64*	F3: TAATACGACTCACTATAGGGGAAGCGTGGTCTGTTTG	dsRNA synthesis
	R3: TAATACGACTCACTATAGGGTGGGTAGGGTTTTGGTA	
*GFP*	F4: TAATACGACTCACTATAGGGCAGTGCTTCAGCCGCTACCC	
	R4: TAATACGACTCACTATAGGGACTCCAGCAGGACCATGTGAT	
*DcCP64*	F5: CCGGAATTCGAGGACAAGAAAGTCGCA	Protein expression
	R5: CCGCTCGAGGTGGTGGTAGTTGTAACTGTAG	

The underline indicates the T7 promoter sequences; the bold underline indicates the sites of restriction enzymes.

## Data Availability

The data presented in this study are available in article or Supplementary Materials.

## Supplementary Materials

The following supporting information can be downloaded at: https://www.mdpi.com/article/10.3390/insects13040353/s1. Figure S1: Analysis of primer amplification efficiency for DcCP64 and GAPDH. Table S1: A summary of the transcriptomes in the different treatments in D. citri. Table S2: A summary of reads mapped to D. citri genome in different treatments. Table S3: Identification of differentially expressed genes in different treatments. Table S4: GO enrichment analysis of DEGs. Table S5: KEGG enrichment analysis of DEGs.
