# Supplementary material for: Silencing of Chitin-Binding Protein with PYPV-Rich Domain Impairs Cuticle and Wing Development in the Asian Citrus Psyllid, Diaphorina citri
Source: Insects. 2022 Apr 2;13(4):353. doi: 10.3390/insects13040353 (PMC9027310; doi:10.3390/insects13040353)
Supplement: Supplementary file 1 [file insects-13-00353-s001.zip › Figure S1.pdf]

Figure S1. Analysis of primer amplification efficiency for *DcCP64* and *GAPDH*.

Primer efficiency of *DcCP64*

		Log10(Ctemplate)	Ct			Average
Undiluted	959.2	2.9819092	24.57	24.42	24.39	24.46
10×	95.92	1.9819092	27.39	27.46	27.46	27.436667
100×	9.592	0.9819092	31.32	31.18	30.87	31.123333
1000×	0.9592	-0.018091	34.51	34.99	34.11	34.536667

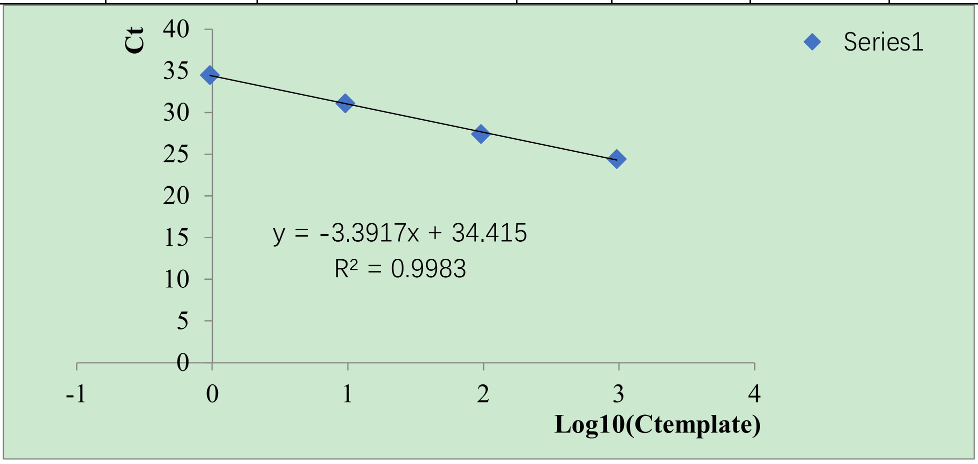

$e = 10^{[-1/slope]} - 1 = 97.24\%$

Primer efficiency of *GAPDH*

		Log10(Ctemplate)	Ct			Average
Undiluted	959.2	2.98190917	23.35	23.18	22.36	22.963333
10×	95.92	1.98190917	25.27	25.52	25.19	25.326667
100×	9.592	0.98190917	28.77	28.73	28.48	28.66
1000×	0.9592	-0.01809083	33.78	33.27	33.22	33.423333

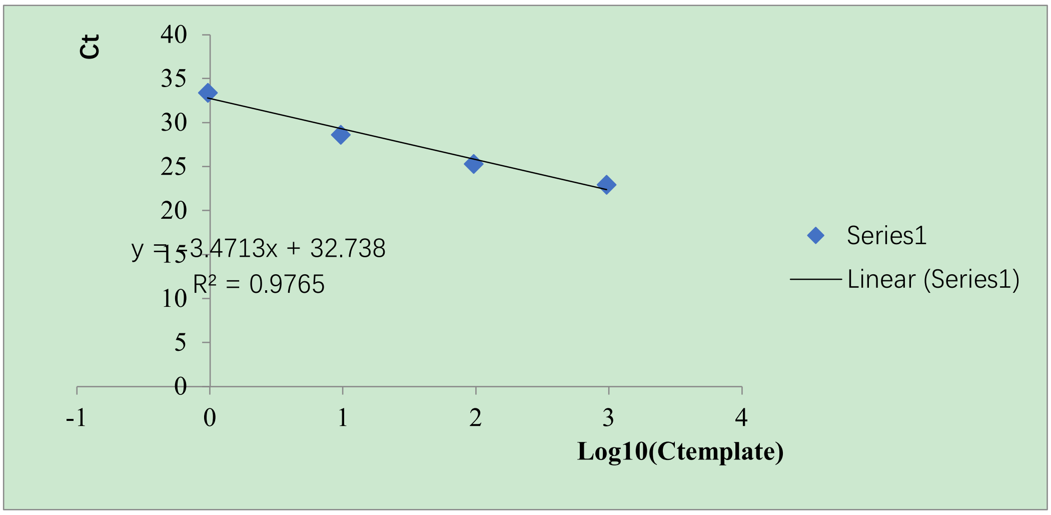

$e = 10^{[-1/slope]} - 1 = 94.13\%$
